# Family Caregiving, Chronic Care and Nursing: Reframing an Unsustainable Care Settlement for the Next 50 Years

**DOI:** 10.1111/jan.70606

**Published:** 2026-04-08

**Authors:** Angela Durante

**Affiliations:** ^1^ Scuola Superiore “S. Anna” Health Science Center Pisa Italy; ^2^ Fondazione CNR/Regione Toscana “Gabriele Monasterio” Pisa Italy

## 1 Introduction: Chronic Disease and the Expansion of Lived Care

There is little doubt that nursing has undergone profound transformation over the past half‐century, expanding its scientific foundations, clinical scope, and professional autonomy. However, it is not only the profession itself that has evolved, but also the very subject of its care: people whose health trajectories are increasingly shaped by complex social, demographic and epidemiological dynamics. Patterns of aging, multimorbidity and long‐term illness have redefined what it means to live with disease, shifting care from discrete clinical events to prolonged, relational, and often uncertain processes. However, healthcare systems have been slower to adapt to these changes. Despite the growing centrality of chronic diseases, care delivery remains largely organized around hospital‐based episodic models, often at the expense of continuity and long‐term support. This misalignment between evolving care needs and persistent system structures has contributed to the progressive relocation of care into domestic and social contexts, where it is largely sustained by patients and those who support them, namely informal caregivers.

Cardiovascular diseases (CVDs) exemplify this transformation. The burden of CVDs has increased markedly since 1990, and most of this burden is attributable to modifiable risk factors (Global Burden of Cardiovascular Diseases and Risks 2023 Collaborators 2025). Despite significant advances in diagnostics, pharmacotherapy, acute care, and prevention, CVDs remain the leading cause of death worldwide. Over time, reductions in mortality have not diminished their impact, but instead transformed them into long‐term conditions, increasing the number of people living with chronic cardiovascular disease and shifting the burden of care from acute events to ongoing management. This transformation has unfolded progressively. Advances in coronary care, pharmacological treatments, and risk factor management have transformed rapidly fatal conditions into survivable, but persistent illnesses. Nursing has been central to this shift. From early coronary care units to contemporary community‐based care, nurses have played a key role in extending survival, supporting self‐care, and translating clinical innovation into everyday practice. At the same time, these achievements have contributed to a new clinical reality: a growing population living longer with complex, chronic, and often unstable conditions that require continuous care beyond hospital settings. This trend is global. Although it is particularly evident in low‐ and middle‐income countries, where most cardiovascular deaths continue to occur (World Health Organization (WHO) 2025), it is also driven by demographic aging and improved survival in multiple areas of disease, including oncology. As longevity increases, so does cardiovascular morbidity, often in the context of multimorbidity and functional decline. Projections suggest that this trajectory will intensify, consolidating CVD as a life‐long condition embedded within everyday life.

In this context, informal care has moved from a supportive element of care to a foundational component of the management of chronic diseases. Informal caregivers (typically family members, but also individuals within the patient's close relational network) provide unpaid, non‐professional support that enables adherence to treatment, symptom monitoring, and everyday functioning. What has changed is not simply the presence of caregiving, but its weight: caregivers are now expected to support complex, prolonged, and clinically relevant care processes that extend far beyond clinical encounters. For nursing, this shift is not peripheral. It redefines the conditions under which care is provided, the feasibility of care plans, and the outcomes of clinical interventions. This commentary advances a central argument: contemporary chronic care is already co‐produced by nurses and informal caregivers, yet this co‐production remains insufficiently recognized, inadequately supported, and increasingly difficult to sustain. Understanding how this situation emerged, how it operates in practice, and how it can evolve is essential to define the role of nursing over the next 50 years.

## 2 From Supporting Role to Structural Dependency

Informal caregivers play an important role in the management of chronic illnesses, particularly in conditions where daily self‐care behaviours are essential to maintain stability and prevent deterioration. Caregiving encompasses a wide range of activities, including symptom surveillance, medication management, coordination of services, and emotional support, often sustained over extended periods. Historically, many of these activities were more closely aligned with professional care and administered within institutional settings. However, several converging factors have progressively shifted this responsibility to the home. The growing number of people who require continuous care, combined with persistent shortages of nursing workers, has made it increasingly difficult to ensure continuity in acute and chronic settings. These challenges are further amplified in rural and underserved areas, where access to ongoing professional support is limited. As a result, continuous care is now provided largely in domestic settings, often without direct supervision and with limited opportunities for ongoing training, reinforcement, or monitoring of the education provided to caregivers. This shift places caregivers in a position where they are expected not only to support care but to maintain it. Keeping CVDs as an example and especially in heart failure, this expectation becomes especially evident. Caregiving requires continuous vigilance: monitoring weight fluctuations, recognizing early signs of fluid retention, managing complex pharmacological regimens, and deciding when clinical deterioration requires escalation. These are not auxiliary tasks, but forms of applied clinical judgement, developed through experience rather than formal training.

For nursing, this shift requires more explicit and systematic engagement with caregiving as a clinical component of care, a position that is increasingly reflected in international guidelines. Assessing caregiver capacity, ensuring the adequacy and retention of education, and identifying early signs of caregiver strain are essential to prevent clinical deterioration. In this sense, caregiver preparedness and sustainability should not be understood as contextual factors but as integral determinants of care quality and patient outcomes. However, this recognition remains only partial in practice. In many health systems (particularly those characterized by familial welfare arrangements), caregivers serve as the primary safety net for people with chronic diseases. What is often described as “family involvement” has evolved into a form of structural dependency, with healthcare systems relying on caregivers to absorb care demands that exceed formal service capacity, especially in the context of shorter hospital stays, outpatient care models and limited resources. Within this framework, the assumption that family members are both available and prepared to assume caregiving responsibilities is rarely questioned. Caregiving is often treated as necessary and, therefore, given, rather than as a role that requires assessment, support, and negotiation. However, this assumption overlooks a critical issue: whether caregivers are truly equipped to support these responsibilities and how this role reshapes their lives, health, and social participation over time.

Evidence from chronic disease management, particularly in cardiovascular conditions such as heart failure, shows that interventions involving both patients and caregivers are associated with improved clinical outcomes, including reduced hospital readmissions and improved quality of life (Buck et al. 2025). Similar patterns are observed in other chronic conditions, such as diabetes, where effective self‐management depends on the consistent integration of treatment regimens into daily life. These findings reinforce the central role of caregivers in supporting complex and ongoing care while also illustrating the extent to which care processes have been redistributed beyond traditional professional boundaries. For nursing, this redistribution has direct clinical implications. It extends the scope of practice beyond patient‐focused interventions to include preparing caregivers to recognize early signs of instability (e.g., fluid retention in heart failure or glycemic fluctuations in diabetes), translating clinical knowledge into sustainable daily routines, and anticipating potential points of failure in home‐based management. However, these responsibilities are often enacted within systems characterized by fragmentation and limited continuity of care. As a result, a persistent gap emerges between what caregivers are expected to manage and the level of support they receive to do so safely and effectively.

## 3 The Contradiction of Informal Caregiving: Essential, Yet Unsustainable

Informal care occupies a paradoxical position within contemporary healthcare systems. It is essential for the functioning of chronic care, but its sustainability is rarely examined from the personal to the community level. Although caregiving can generate relational meaning and support patient outcomes, it is also associated with substantial physical, emotional, and social costs. In many contexts, even high‐income countries, caregiving remains strongly gendered, with women disproportionately assuming caregiving roles. It is also associated with reduced labor market participation, including fewer working hours and earlier retirement, generating measurable economic consequences (Organisation for Economic Co‐operation and Development (OECD) 2025). These patterns demonstrate that caregiving is not simply a private matter, but a structural component of both the health and economic systems.

At an individual level, caregivers frequently experience fatigue, psychological distress, financial stress, and social isolation. The cumulative impact of these demands has been conceptualized as a burden on caregivers, reflecting the growing recognition of caregiving as a site of vulnerability rather than a site of only resilience. Importantly, this burden is not incidental but structurally produced, emerging from the progressive transfer of care responsibilities to families without corresponding and sustained formal support. Even in countries where community‐based and family‐oriented care systems are relatively well developed, significant gaps remain. The absence of continuous responsive support (particularly outside standard service hours), combined with the weakening of informal community networks in contemporary societies, often leaves caregivers without viable alternatives.

Moving to a more explicitly nursing‐focused perspective, these dynamics translate into concrete clinical consequences, often preceding or occurring alongside social ones. When caregiving capacity is exceeded, the deterioration becomes frequently visible through acute events. In chronic conditions, this can manifest as delayed responses to worsening of symptoms, inconsistent medication management, or difficulty maintaining dietary and fluid restrictions. Such events are commonly interpreted as issues of adherence or disease progression; however, they may instead reflect the limits of caregiving capacity. Emergency departments often become the point at which these limits surface. Patients present with advanced symptoms that might have been earlier managed under different conditions, while caregivers arrive after prolonged periods of stress and uncertainty. Without explicit recognition of caregiving as a determinant of these outcomes, clinical responses risk addressing symptoms without addressing their underlying causes. In the post‐pandemic period, several potential strategies have emerged to strengthen nursing support beyond hospital settings. In some contexts, this has involved expanding family and community nursing models; in others, integrating these approaches with telemedicine. Although these developments have shown promise, telemedicine has at times been interpreted as a comprehensive solution to workforce shortages and the growing burden of chronic diseases. However, in practice, many of these innovations have remained localized or episodic, demonstrating effectiveness in specific settings without achieving the level of scalability required for system‐wide impact. As a result, their potential to support sustained collaboration between nursing and informal caregiving remains only partially realized.

## 4 Clinical Uncertainty and the Expansion of Nursing Responsibility

The challenges associated with caregiving are further compounded by the nature of chronic illness trajectories, which are often unpredictable and characterized by periods of relative stability interspersed with acute deterioration. Throughout this review, chronic cardiovascular diseases have been used as a guiding example, with heart failure particularly clearly illustrating this uncertainty. Despite mortality rates comparable to many cancers, heart failure is still often perceived as a manageable disease rather than a life‐limiting one. This misperception contributes to delayed self‐care engagement, limited recognition of clinical deterioration, and postponed or unaddressed palliative care discussions, ultimately reducing preparedness for end‐of‐life transitions among both patients and caregivers.

At the same time, these considerations extend beyond cardiovascular disease and are applicable to a wide range of chronic conditions, reflecting shared structural and clinical dynamics rather than disease‐specific features. In addition, chronic illness does not exclusively affect older populations. Although aging remains a major driver of prevalence, long‐term conditions increasingly span the entire life course. Within cardiovascular diseases, congenital heart conditions exemplify forms of chronicity that begin early in life and require sustained management over decades. This broader perspective highlights the need to conceptualize caregiving not as a time‐limited or age‐bound phenomenon, but as a long‐term, evolving process that adapts to changing clinical and life circumstances. For nursing, this requires a shift toward anticipatory and trajectory‐based care, in which the timing of interventions, the preparation of caregivers, and the recognition of transitions (including end‐of‐life phases) become integral components of clinical practice rather than reactive responses to acute deterioration.

In this context, caregivers are required to interpret symptoms, manage complex clinical information, and make decisions under conditions of uncertainty. They function as intermediaries between patients and healthcare systems, often assuming responsibilities that extend beyond traditional expectations. For nursing, this uncertainty translates into an expanded responsibility that remains only partially formalized. Nurses are required not only to manage clinical conditions but also to anticipate instability, support caregiver decision‐making, and prepare families for trajectories that are often difficult to define. This includes initiating conversations about deterioration and end‐of‐life transitions earlier than is commonly practiced. However, this expanded role is frequently constrained by organizational models that prioritize episodic care over continuity. As a result, nurses can recognize the limits of caregiving capacity without having the structural means to intervene effectively. This tension between clinical awareness and system constraint is likely to become more pronounced as the prevalence of chronic diseases increases.

## 5 Changing Families and the Erosion of Assumed Care Capacity

The sustainability of informal caregiving is further challenged by broader social and demographic transformations. Family structures have become more diverse and less predictable, characterized by smaller household sizes, geographic dispersion, and changing patterns of kinship and partnership. At the same time, increased participation in the workforce and economic pressures have reduced the availability of time and resources for caregiving, especially for women. While these changes reflect important social progress including greater gender equity and individual autonomy, they also expose the fragility of care models based on assumptions of family availability. The expectation that families will continue to absorb increasing care demands without substantial support is increasingly untenable. Looking ahead, these trends suggest not only a reduction in the number of available caregivers, but also an increase in the complexity of caregiving arrangements, with care distributed across fewer individuals, over longer periods, and within contexts full of competing demands.

In response to these transformations, many health and social systems have witnessed a growing reliance on paid caregivers, often migrant workers from lower‐income countries. Initially employed to support household tasks, these roles have progressively expanded to include direct care of individuals with complex health needs. While this shift has partially compensated for gaps in informal caregiving, it introduces additional challenges related to cultural differences, language barriers (particularly in medication management), and potential changes in patients' daily habits, including diet and lifestyle. At the same time, this workforce remains relatively underexplored within nursing and health services research, with limited evidence on training needs, integration into care teams, and impact on patient and caregiver outcomes. Although the regulatory and contractual aspects of these roles remain variable and, in some contexts, insufficiently formalized, their presence is becoming increasingly central within contemporary care arrangements. On a nursing perspective, this evolving landscape requires engagement not only with patients and family caregivers, but also with a broader and more heterogeneous caregiving workforce. This further reinforces the need for anticipatory and trajectory‐based care, in which coordination, education, and support extend across all those involved in sustaining care beyond clinical settings.

Addressing these challenges requires a shift in the way caregiving is conceptualized within nursing. Caregiving must be recognized as a core domain of practice, research, and policy engagement. This involves treating caregiving as a clinical variable to be assessed and integrated into care planning, recognizing it as a site of inequality shaped by social and economic conditions, and understanding it as a reflection of broader decisions about resource allocation and responsibility. Therefore, the role of nursing is not limited to supporting caregiving but extends to shaping how caregiving is organized, sustained, and integrated into care systems.

## 6 Conclusion: Reframing Caregiving for the Next 50 Years of Nursing

Over the past 50 years, nursing has played a central role in transforming acute, often fatal conditions into chronic, manageable diseases. This progress has extended survival and reshaped the experience of illness. However, it has also contributed to a fundamental reconfiguration of care, in which a substantial proportion of clinical work is now sustained outside formal healthcare settings. This commentary argued that contemporary chronic care is no longer delivered solely within healthcare systems but is co‐produced through the interaction between nursing and informal caregiving. While this model has enabled the management of increasingly complex and prolonged conditions, it has also generated a structural imbalance: caregiving is essential, yet insufficiently recognized, inadequately supported, and implicitly assumed to be available. The limits of this arrangement are already visible in clinical practice. When caregiving capacity is exceeded, the consequences emerge as delayed responses to deterioration, increased reliance on emergency services, and unmet care needs. These are not isolated failures, but indicators of a system that depends on resources it does not actively sustain. Looking ahead, demographic change, evolving family structures, and workforce constraints will further challenge the availability and feasibility of informal caregiving. In this context, nursing cannot remain positioned solely as a profession that supports caregiving, but must actively shape how caregiving is assessed, integrated, and sustained within care systems. For the next 50 years tree key points need to be considered to improve nursing care for people with chronic illnesses.

First, caregiving must be systematically assessed as a clinical variable. This requires moving beyond informal or implicit evaluation toward structured and documented assessment processes. A critical step in this direction is the development and routine use of caregiver‐reported outcome measures, which remain insufficiently standardized and validated despite their potential to capture caregiver capacity, preparedness, and strain over time. These measures should be integrated into clinical pathways alongside patient‐reported outcomes, informing care planning, discharge decisions, and risk stratification. At a system level, caregiver assessment should be embedded within electronic health records and continuity‐of‐care models, ensuring that caregiving capacity directly influences clinical decision‐making. More broadly, this shift aligns with value‐based healthcare approaches, where outcomes extend beyond patients to include the sustainability of caregiving arrangements, making caregiver support a measurable component of care quality and system performance.

Second, nursing interventions must extend beyond patient education to include structured and longitudinal support for caregivers. This involves shifting from one‐time instruction (often still delivered at discharge) to ongoing, adaptive support models. Practical strategies include scheduled follow‐up contacts, reinforcement sessions focused on key self‐care skills, and the use of digital tools to monitor symptom interpretation and care practices over time. Early identification of caregiver strain should trigger tailored support interventions, rather than reactive responses to clinical deterioration. From a policy perspective, this requires recognizing caregiver education and support as core nursing activities, with dedicated time, resources, and accountability indicators, rather than as informal extensions of patient care. Despite some countries having already introduced discharge clinics and virtuous collaborations among nurses from different healthcare settings, these remain isolated best practices still not implemented equally in all the countries.

Third, nursing must take a more active role in coordinating increasingly complex care ecosystems. As caregiving expands to include family members, paid caregivers, and community‐based actors (like third sector volunteers or patient‐caregivers associations), nurses are uniquely positioned to ensure coherence, continuity, and safety across these interfaces. This includes actively involving paid caregivers (including migrant care workers) in education and communication processes, clarifying roles within care plans, and facilitating structured information exchange between formal and informal care providers. At an organizational level, this calls for integrated care models in which nursing assumes a recognized coordination function across settings, supported by policies that formally acknowledge and regulate the contribution of diverse caregiving actors.

In conclusion, the next 50 years of nursing will depend not only on its ability to manage chronic illness, but on its capacity to actively shape and sustain the conditions under which care management occurs. This includes recognizing caregiving as a clinical and organizational determinant, integrating it into assessment, care planning, and system design, and ensuring that it is supported through adequate resources, structures, and policies. Without this shift, the progress achieved in extending life risks being undermined by the fragility and invisibility of the care arrangements on which it increasingly depends.

## Author Contributions

A.D.: Conceptualization, writing – review and editing.

## Funding

The author has nothing to report.

## Conflicts of Interest

The author declares no conflicts of interest.

## Data Availability

Data sharing not applicable to this article as no datasets were generated or analysed during the current study.

## References

[jan70606-bib-0002] Buck, H. G. , A. Durante , C. Howland , et al. 2025. “Examining Heart Failure Informal Care Partners Using Person and System Levels and Domains: A Meta‐Synthesis.” Western Journal of Nursing Research 47, no. 4: 261–281. 39888665 10.1177/01939459251314716

[jan70606-bib-0003] Global Burden of Cardiovascular Diseases and Risks 2023 Collaborators . 2025. “Global, Regional, and National Burden of Cardiovascular Diseases and Risk Factors in 204 Countries and Territories, 1990‐2023.” Journal of the American College of Cardiology 86, no. 22: 2167–2243. 40990886 10.1016/j.jacc.2025.08.015

[jan70606-bib-0004] Organisation for Economic Co‐operation and Development (OECD) . 2025. Health at a Glance 2025: OECD Indicators. OECD Publishing. https://www.oecd.org/en/publications/health‐at‐a‐glance‐2025_8f9e3f98‐en/.

[jan70606-bib-0005] World Health Organization (WHO) . 2025. “Cardiovascular Diseases (CVDs).” https://www.who.int/news‐room/fact‐sheets/detail/cardiovascular‐diseases‐%28cvds%29.

